# Beliefs About Children’s Emotions in Chile

**DOI:** 10.3389/fpsyg.2020.00034

**Published:** 2020-01-30

**Authors:** Amy G. Halberstadt, Dejah Oertwig, Enrique H. Riquelme

**Affiliations:** ^1^Department of Psychology, North Carolina State University, Raleigh, NC, United States; ^2^Facultad de Educación, Universidad Católica de Temuco, Temuco, Chile

**Keywords:** emotion beliefs, Mapuche, Chile, teacher beliefs, parent, emotion regulation, nature, fear

## Abstract

To learn more about Chilean emotional beliefs related to emotion development, 271 Mapuche and non-Mapuche parents and teachers in urban and rural settings reported their emotion beliefs using a questionnaire invariant in the Chilean context (Riquelme et al., in press). Included are six beliefs previously found to resonate across three United States cultures (i.e., beliefs about the value and cost of certain emotions; control of emotion; knowledge of children’s emotion; manipulation of emotion; and emotional autonomy), and five others distinctive to the indigenous people of this region (i.e., value of being calm; controlling fear specifically; interpersonality of emotion; learning about emotion from adults; and regulation through nature). MANOVAs were conducted to examine these beliefs across culture (Mapuche, non-Mapuche), role (parent, teacher), and geographical location (rural, urban). For United States-derived beliefs, there were no main effects, although two interactions with culture by role and location were significant. For all five Mapuche-generated beliefs, there were significant main effects for culture, role, and location. Results highlight both similarities and differences in beliefs across cultures, roles, and geographical location. Implications for the Chilean context include the importance of non-Mapuche teachers’ sensitivity to the values and emotion-related beliefs of Mapuche families. Implications for the global context include an expanded view of emotion-related beliefs, including beliefs that children can control fear and be calm, that emotion-related values include attending to the needs of others, and that two ways of controlling emotion are through learning by listening to/watching elders, and by being in nature.

## Introduction

Beliefs about emotions are thought to be important in influencing individuals’ own behaviors and how they respond to others (Gottman et al., 1996; Halberstadt et al., 2013; Tamir and Bigman, 2018; Ford and Gross, 2019). By beliefs we rely on Goodenough (1963) and McGillicuddy-De Lisi and Sigel (1995) who describe beliefs as propositional statements about the world assumed to be valid or true. As with other beliefs, beliefs about emotions serve as guides for decision-making and evaluating behaviors of self and others, and are sometimes so strongly accepted as fact that their true nature as personal belief is not noted (McGillicuddy-De Lisi and Sigel, 1995; Richardson, 1996). A number of emotion beliefs have been identified, including whether emotions are of value, adults should guide emotions or give autonomy to children to figure out their own emotional lives, emotions are authentic or manipulative, emotional behavior is contagious or residue, children can control their emotions, and emotions are (are not) interrelated with learning (Hyson and Lee, 1996; Dunsmore et al., 2009; Parker et al., 2012; Swartz and McElwain, 2012; Halberstadt et al., 2013; Bächler and Pozo, 2016; Di Giunta et al., 2017; Hart and DiPerna, 2017; Hagan et al., 2020).

The origin of emotion beliefs is most likely situated in the cultural context (Mesquita and Frijda, 1992; Cole and Tan, 2007; Mesquita and Albert, 2007; Dunsmore and Halberstadt, 2009; Harkness et al., 2011), and as evidence, emotion beliefs often vary by culture. Importantly, they almost always reflect and maintain the existing values, norms, and customs of that community of people (Harkness and Super, 1992; Friedlmeier et al., 2015; Tamir et al., 2016; Raval and Walker, 2019). Because beliefs often guide behaviors (Dennis and Halberstadt, 2012; Lozada et al., 2016; Hagan et al., 2020), the everyday beliefs that people hold can help us to understand more about similarities and differences in emotion socialization across cultures.

Differences can also occur for cultures co-existing within a region. For example, in Nepal, the Tamang participate in Tibetan Buddhism, with its relatively egalitarian orientation, emphasis on compassion, and willingness to share with others. When socializing children, they tend to ignore/minimize reasons for anger, and instead discuss and nurture children’s experiences of shame. In this way, they promote their ideal of socially graceful, non-angry children. In contrast, the Brahman, with their hierarchically privileged position in the Hindu caste system, tend to nurture anger while ignoring shame in their socialization practices (Cole et al., 2006). Thus, for cultures that appear initially similar (i.e., values or location), some degree of difference can emerge, and these differences may influence children’s outcomes.

Studying emotion beliefs across cultures not only informs us about other cultures but also gives us insight into our own cultural beliefs and values, as well as the opportunity to reflect on or change the beliefs and values we want to inculcate in our young. Recognizing the connections from particular beliefs to behavior, and then to outcome, allows adults to better consider how to socialize toward compassion or power in the young of their own cultures (Tamir et al., 2016).

Although emotion-related beliefs have been investigated in the United States, Europe, and Asia, the study of culture and emotion is still incipient in South America with its heterogenous cultures, and the meeting of various original and dominant occidental cultures. Our work answers the call to investigate more of the cultural possibilities in emotion-related values and experiences across the world (Raval and Walker, 2019), and to expand our understanding of the role of different adults and geographies in the development of emotion-related beliefs. We were particularly interested in exploring both similarities and differences across cultures, and we had the opportunity to include one of the oldest original cultures in the Americas, which is also a culture that has retained many of its customs and beliefs. For this study, we examined two cultures living side-by-side in Chile and their beliefs about children’s emotions. Our goals in doing so were to explore similarities or differences in beliefs across the Americas, within cultures co-existing within a region, and by role (parent vs. teacher) and geography (rural vs. urban). We begin by describing the Mapuche briefly and why the study of their beliefs is so compelling.

### The Mapuche

The Mapuche are the original people living in Southern Chile and Argentina (Mapuche means “people of the land”). They have managed to live continuously in their region, having withstood attempted invasions by the Incas and holding off Spanish invaders for over 300 years. When the newly formed countries of Chile and Argentina joined forces, they were able to finally subdue the Mapuche. Relations between the Mapuche and the Chilean state since then have been characterized by a latent conflict marked by the struggle for recovery and protection of the territories known to the Mapuche as well as recognition in social and educational domains. Despite or perhaps because of these tensions, the Mapuche have been able to maintain a good portion of their customs and cultural beliefs, particularly in the rural areas, without becoming fully assimilated into mainstream Chilean society.

A deep respect for the environment as the good way to live (“*Küme Mongen*” in Mapuzungun; de la Cuadra, 2015) is part of a central belief in the oneness of all things. The Mapuche believe that they and nature are part of the same whole (Mariqueo and Calbucura, 2016). The strong sense of responsibility for nature is evident in the Mapuche fight to protect the land in various ways for over 400 years, including today, as the Mapuche continue to resist the subjugation of their culture and the land (Brady, 2018; Youkee, 2018).

The current emotion-related beliefs of the Mapuche likely emerge from their ancestral knowledge, culture, and history. In addition to the centrality of nature and its protection, or perhaps because of it, Mapuche history has been intricately intertwined with war for many centuries now. The feature of fearlessness may have enabled the Mapuche to withstand both Inca and Spanish encroachment over the centuries, or may have resulted from the absolute need to protect and defend the land (Oertwig et al., 2019). Additionally, the well-established vertical hierarchy of the Mapuche supports the passing down of traditional, emotional beliefs through the values of respect for and learning from elders, as is true for other societies that focus on power as an important socially relevant dimension (Matsumoto, 1996; Triandis and Gelfand, 1998; Hofstede, 2001). In these ways, Mapuche values are likely to vary from, and, indeed, may clash with the values expressed (implicit or explicitly) in non-Mapuche Chilean culture, which tends to be more based in occidental, Western-dominated values.

However, some Mapuche have succumbed to the devaluation and discrimination imposed by Chilean dominant culture (largely Spanish and German origin in this region) and the adoption of North American economic principles, and have chosen to assimilate into the wider culture. Inevitably, as the cultures have lived in some proximity over the centuries, beliefs have been shared and have intermingled. Thus, although we focus on differentiating Mapuche beliefs and non-Mapuche beliefs, partly in order to highlight the value of these oppressed people’s beliefs, it is important to note that the two cultures may have moved toward each other in various beliefs over time.

### Parent and Teacher Roles

Parents are important in guiding emotion-related values and beliefs in many cultures, and do so via multiple behavioral pathways (Dunsmore and Halberstadt, 1997; Eisenberg et al., 1998a, b). Empirical evidence is abundant. For example, parents who believe that emotions are dangerous or problematic are more likely to mask their emotional expressions so that their children do not assume that expressiveness is appropriate or imitate such expressions themselves (Halberstadt et al., 2008; Dunsmore et al., 2009), compared with parents who do not have this view of emotion. Additionally, parents seeking to cultivate the emotion of gratitude are more likely to place their children in social niches that support experiencing gratitude (Rothenberg et al., 2017).

Teachers are also powerful socializers and important to children who spend over 1000 h per year in school. The student-teacher relationship greatly impacts a student’s school experience (Garner and Mahatmya, 2015) and is best fostered through the student’s adherence to teacher expectations (Lane et al., 2004). Because the school context necessarily involves many children and few adults, teacher beliefs might vary from parents, for example, regarding children’s ability to control their emotion, importance of controlling their emotion, and ways in which emotions might support or deter knowledge acquisition (Bächler and Pozo, 2016).

A lack of congruence between Mapuche parent and non-Mapuche teacher beliefs has also been thought to be responsible for some of the problematic outcomes for Mapuche children in school (Riquelme et al., 2016, 2017). First, more occidentally trained, western-centered teachers may privilege a curriculum that focuses on learning by reading and writing, and through children’s active, emotional participation, whereas Mapuche culture emphasizes learning in and from the natural world, and values children quietly listening and observing adults as the appropriate way to learn forms of knowledge that cannot be directly experienced (Quilaqueo and Torres, 2013). Second, teachers learn and are certified within majority-culture training programs with a dominant set of expectations about behavior, which can create invisible disadvantages for minority children. Such beliefs about children’s emotions may be implicit (i.e., fall into the hidden curriculum), with teachers expecting behaviors they have learned to identify in their programs, and problematizing behaviors that might only reflect minority values. For example, Mapuche children learn not to talk to adults but to listen to them, and also rural children rarely encounter strangers, with whom they would talk with even less. Thus, they often fail language assessments with strange testers. In this way, they are frequently but falsely diagnosed with emotional and behavioral disorders (Gutierrez Saldivia, 2018), suggesting that disproportionality may start in our beliefs and practices rather than in the children (Riquelme et al., 2017).

### Geography

Geographic location affects how people relate to their environment, and indeed, influences their communities, cultures, and economies, as well as interpersonal relations with and across space and place (Davidson et al., 2005). Given the centrality of nature in Mapuche thinking, we thought that the geographical locations in which the Mapuche live may be particularly relevant for maintaining their emotion-related beliefs. The urban-rural location is an important distinction because where people live affects whether family members spend a lot of time outdoors or find themselves together in a small (or large) dwelling, and socialize with others who are strangers or known others. The type of terrain also influences how time is spent (e.g., collecting food from fields or in an money-based economy, with a grocery store nearby or far away, with few or many hazards to navigate though), types of work (e.g., agriculture versus trade, commerce, or service) and forms of recreation (e.g., playing in fields or woods versus reading a book, engaging in technology, or team sports). Geography has implications for family harmony, children’s independence, responsibility, and proximity to nature, and all of these cultural patterns can, in turn, influence groups’ emotional patterns (Harkness and Super, 1992; Herron and Skinner, 2012).

Although the effects of geography on emotion-related beliefs have not been well-explored, it is easy to imagine that a rural, agrarian lifestyle with small homes, well-spaced so as to support cultivating animals and crops, might be emotionally different than city life, with apartments close together, children having less independence or responsibility outside their homes, and more asphalt, cars, people, and traffic. We thought that rural Mapuche living in traditional, agrarian communities might have more connection to the land and their source of spirituality and wholeness, particularly for beliefs embedded in the particularities of a geographical location. In contrast, we thought that urban Mapuche living in cities, with exposure to the diverse beliefs of others, might have difficulty holding onto ancestral cultural beliefs as well as their connection to the land.

### The Current Study

Our goals were to explore the similarities and distinctiveness in socialization beliefs about emotion. We had three aims. First, we wanted to learn whether beliefs identified in three cultures within the United States would be relevant in another county and in a culture distinctive from the cultures in which the scales originated. Although (or because) there are many countries, geographically separating the United States and Chile, which were created with different European influences, similarities would help inform us regarding the widespread relevance of these beliefs across the Americas. Second, we wanted to learn more about the emotion-related beliefs in the distinctive culture of the Mapuche, and to introduce Mapuche beliefs to other cultures, because these beliefs, once articulated, could be meaningful for other South or North American cultures and, indeed, worldwide. Third, because “culture” involves many components, we wanted to explore culture as (a) the different groups of a region, focusing on Mapuche versus non-Mapuche as one distinction that the people in Chile find meaningful, (b) the defining roles of the participants, focusing on whether participants are parents or teachers, which implies different responsibilities with children and engagement with the dominant ideology regarding educating children, and (c) geography, focusing on the rural versus urban location of the participants, which reflects a host of physical and community influences.

To examine Chilean emotional beliefs about children, 271 Mapuche and non-Mapuche parents and teachers completed a questionnaire (Riquelme et al., in press). The questionnaire includes six subscales derived from the PBACE Questionnaire (Halberstadt et al., 2013) and five new subscales derived from Mapuche values (Quilaqueo, 2006; Oertwig et al., 2019). Following best practices for cross-cultural measurement, we chose a questionnaire that had achieved configural, metric, and at least partial scalar invariance across Mapuche and non-Mapuche participants (Riquelme et al., in press).

Although cross-cultural work often focuses on finding differences, we felt it would be presumptuous to assume differences in the six subscales from the United States, as any hypotheses would not be theoretically derived. We did, however, hypothesize significant cultural differences between the Mapuche and non-Mapuche Chileans for the Mapuche-derived beliefs. We thought that parents’ beliefs would be more differentiated across cultures than teachers, because the dominant educational ideology might have influenced teachers’ beliefs to be more similar across cultures. We also thought that rural participants might be more differentiated than city participants for two reasons. In the city, the constant engagement with heterogeneity of beliefs might ultimately lead to greater homogeneity in mean scores across the cultures, as the populations assimilate toward each other. Additionally, rural participants might more successfully retain the beliefs of their heritage, particularly the beliefs that are embedded in interconnections to nature and place, which are more present in rural life. To assess these general research questions and hypotheses, we analyzed beliefs using MANOVAs that included culture (Mapuche, non-Mapuche), role (parent, teacher), and geography (urban, rural).

## Materials and Methods

### Participants

The 271 participants included 106 Mapuche adults (77 parents and 29 teachers [82.1% female; *M* = 33.64 years, *SD* = 8.53, range = 18–57]) and 165 non-Mapuche adults (92 parents and 73 teachers [86.1% female; 38.7% teachers; *M* = 34.70 years, *SD* = 10.45, range = 19–62]), living in the Araucanía region in Chile.

As the largest of the original populations of Chile, the Mapuche comprise almost 13% of Chileans, but 34.3% of the people living in the Araucania region (Instituto Nacional de Estadísticas, 2018). Araucanía is a relatively southern part of Chile known for its lakes, rolling hills, temperate rainforest, and active volcanoes. The region spans the width of Chile, from the Pacific coast to the Andes Mountains. In recent years the Chilean population, like much of the world, is migrating to urban centers, however, the Mapuche are resisting this pattern more so then their non-Mapuche neighbors. In this sample, 60.6% of Mapuche and 12.5% non-Mapuche were living in rural sectors (e.g., farms or small towns near farms).

### Procedure

This study was carried out in accordance with APA recommendations of standards for research and was approved by the research ethics committee of Universidad Católica de Temuco. All participants gave written informed consent in accordance with the Declaration of Helsinki. A team of prospective and current teachers, including many who were first generation in college, recruited teachers from the schools where they had taught or with whom they had contacts, and also parents via the same type of snowball sampling. Because few Mapuche speak Mapuzungun in daily life (e.g., Oertwig et al., 2019 report that all 22 of their informants preferred Spanish when given the choice, although they often referred to concepts specific to Mapuche life in Mapuzungun), the questionnaire was provided in Spanish. We note that although illiteracy is about twice as frequent among indigenous versus non-indigenous adults in Chile, rates are dropping dramatically with each age group; less than 3% Mapuche in the participating age groups are unable to read Spanish (Ministerio de Desarrollo Social y Familia, Encuesta CASEN, 2017). Data were collected in 2016 when there was some tension surrounding land practices, but comparative peace relative to the stressors engendered by events between 2018 and the present.

### Measures

#### Cree-Emociones Cuestionario (Riquelme et al., in press)

This questionnaire includes 11 scales assessing emotion-related beliefs. All scales demonstrate configural, metric, and at least partial scalar invariance with Mapuche and non-Mapuche Chilean participants. Table 1 includes scale names and item examples; the full questionnaire and scoring can be found in the Supplementary Materials.

**TABLE 1:** Descriptions of the emotion-related beliefs.

**Beliefs**	**# of items**	**Examples**		**α**
		**Spanish**	**English Translation**	
**United States-generated**				
Value of anger	3	Es útil para los niños sentir enojo a veces	It is useful for children to feel angry sometimes	0.50
Positivity is costly	4	Cuando los niños están muy felices, pueden salirse de control	When children are too happy, they can get out of control	0.65
Children can control	5	Los niños pueden controlar lo que muestran en sus rostros	Children can control what they show on their faces	0.70
Emotions are manipulative	4	Los niños a veces actúan tristes solo para obtener atención	Children often cry just to get attention	0.74
Know children’s feelings	3	Los padres deberían alentar a sus niños a decirles todo lo que están sintiendo	Parents (teachers) should encourage their children to tell them everything they are feeling	0.71
Autonomy	3	Usualmente es mejor dejar al niño que maneje sus sentimientos negativos por sí solo	It’s usually best to let a child work through their negative feelings on their own	0.56
**Mapuche-generated**				
Control of fear	6	Parte del crecimiento es aprender a no sentir miedo	Part of growing up is learning not to be afraid	0.76
Calm child	7	Estar tranquilo es clave para el control de las emociones.	Being calm is key to the control of emotions	0.79
Kumeche	4	Los niños deben estar atento a las necesidades de los otros	Children must be attentive to the needs of others	0.68
Emotion through observation	4	Los niños aprenden a regularse emocionalmente escuchando a los adultos	Children learn to regulate their emotions by listening to adults	0.71
Regulation through nature	8	La naturaleza puede ayudar a los niños regular sus expresiones emocionales	Nature can help children to regulate their emotional expressions	0.87

In this questionnaire, items representing six beliefs from the Parents’ Beliefs about Children’s Emotions (PBACE, Halberstadt et al., 2013) had been translated into Chilean Spanish and back-translated into English, and revised until translators were satisfied that the Spanish items maintained the deep structure of the original items. The word *parents* was replaced with *adults*, with one exception, so as to be appropriate for both parents and educators. PBACE beliefs were: Value of Anger, Positivity is Costly, Children can Control (their emotions), (children’s) Emotions are Manipulative, Know Children’s Feelings, and Autonomy.

Five beliefs identified within the Mapuche Chilean context had also been identified from semi-structured interviews with Mapuche mothers and elders about their emotion-related values and experiences of children in schools as well as general cultural knowledge (Quilaqueo and Torres, 2013; Oertwig et al., 2019). Items representing these beliefs were reviewed by Chilean parents and teachers for appropriate comprehension following within-language back-translation techniques (Halberstadt et al., 2013; Riquelme et al., in press). These beliefs are: (importance of) Controlling Fear, (importance of) Being Calm, (importance of) Kumeche, Emotion through Observation, and Regulation through Nature. *Kumeche* is a central way of being with and respecting others in Mapuche thinking and can best be described as solidarity, and being attentive, kind, compassionate, and empathetic with others (Quilaqueo, 2006). In some ways, Kumeche represents the emotion ideals organized around being a “good” person in relation to the community. Thus, Kumeche is an interpersonal way of being that supports harmonious interactions.

For all items, participants were asked to choose the item that best fit what they thought, using a scale from one (completely disagree) to six (completely agree).

## Results

### Beliefs From the United States

**FIGURE 1:**
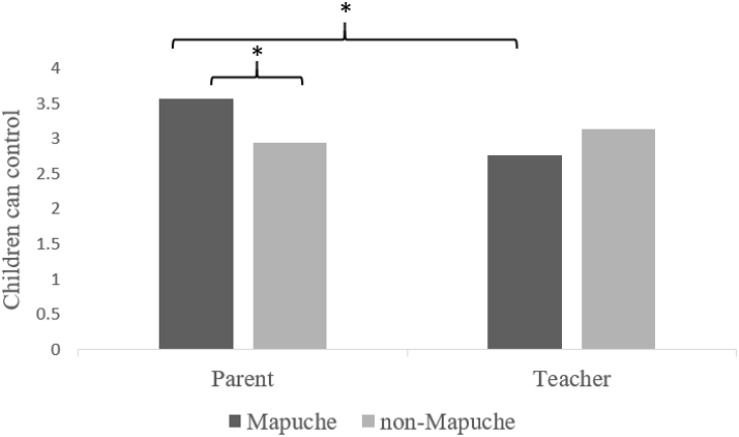
Culture, role, and belief about whether children can control their emotions. *F*(1,270) = 4.98, *p* < 0.05, partial η^2^ = 0.019. ^∗^*p* < 0.01.

A three-way MANOVA was run with three independent variables – culture, role, and geography- and six dependent variables (i.e., beliefs). There were no significant main effects on the combined dependent variables (Table 2). There was, however, an interaction between culture and role (*F*(6,265) = 2.29, *p* < 0.05, η^2^ = 0.053). Follow-up analyses of the six beliefs revealed only one significant interaction; this was for beliefs about children’s abilities to control their emotions. When comparing across culture, Mapuche parents (*M* = 3.58) believed more strongly than non-Mapuche parents (*M* = 2.95) that children can control their emotions, 0.63 (95% CI 0.22 to 1.04) *p* = 0.003, whereas Mapuche and non-Mapuche teachers were in relative agreement (*M*s = 2.77, 3.15, respectively) with non-significant mean score differences, 0.38 (95% CI −0.41 to 1.16) *p* = 0.350. When comparing across role, Mapuche parents (*M* = 3.58) believed more strongly than Mapuche teachers (*M* = 2.77) that children can control their emotions, 0.81 (95% CI 0.27 to 1.35), *p* = 0.003; non-Mapuche parents and teachers (*M*s = 2.95, 3.15, respectively) were in relative agreement, 0.20 (95% CI −0.51 to 0.90), *p* = 0.580 (Figure 1).

**TABLE 2:** Univariate comparisons for the United States-generated beliefs.

	***M* (*SD*)**	
**Culture (combined effect)**	**Mapuche**	**Non-Mapuche**
Value of anger	4.13 (1.14)	3.79 (1.13)
Positivity costly	3.51 (1.30)	3.26 (1.12)
Children can control	3.22 (1.24)	3.00 (1.04)
Emotions are Manipulative	3.46 (1.30)	3.25 (1.30)
Know children’s feelings	5.09 (1.18)	5.19 (1.11)
Autonomy	2.93 (1.16)	2.66 (1.12)

*Participants responded to items using a Likert scale ranging from one (totally disagree) to six (totally agree). The main effect for culture was non-significant; *F*(6,265) = 2.09, *p* = 0.06, η^2^ = 0.05. Because we had no hypotheses and the sample was reasonably large, we could not justify the risk of Type 1 errors incurred by further analyses.*

**FIGURE 2:**
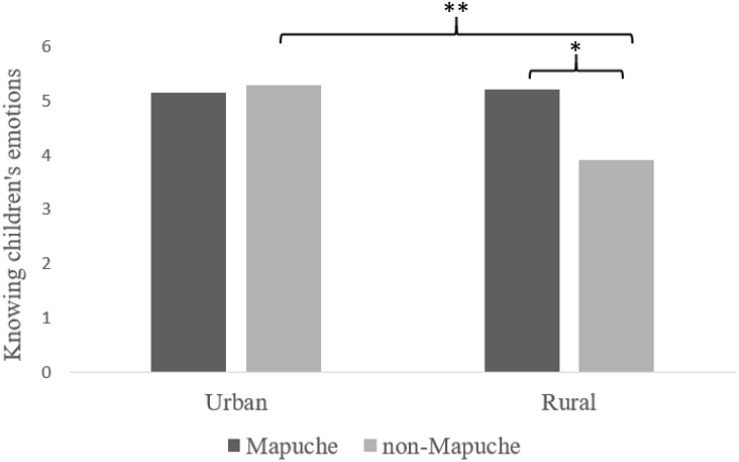
Culture, locale, and the belief about the importance of knowing children’s feelings. *F*(1,270) = 9.98, *p* < 0.01, partial η^2^ = 0.038. ^∗^*p* < 0.05, ^∗∗^*p* < 0.01.

There was also a significant interaction between culture and location (*F*(6,265) = 2.45, *p* < 0.05, η^2^ = 0.056). Follow-up analyses revealed only one significant interaction; this was for the importance of knowing children’s feelings. When comparing cultures, Mapuche and non-Mapuche city-dwellers (*M*s = 5.17, 5.31, respectively) shared relatively similar beliefs, 1.28 (95% CI −0.26 to 0.54) *p* = 0.48, whereas rural Mapuche (*M* = 5.22), and non-Mapuche (*M* = 3.93) differed in importance of knowing children’s feelings, 1.27 (95% CI 0.49 to 2.09) *p* = 0.002. When comparing geography, urban and rural Mapuche (*M*s = 5.17, 5.22, respectively) shared similar beliefs, 0.05 (95% CI −0.50 to 0.59), but urban non-Mapuche (*M* = 5.31) and rural non-Mapuche (*M* = 3.93) differed, yielding a mean difference of 1.38 (95% CI 0.67 to 2.09), *p* < 0.001 (Figure 2).

### New Identified Beliefs From the Mapuche

A three-way MANOVA was run with three independent variables – culture, role, and geography – and five dependent variables (i.e., beliefs). The main effects on the combined dependent variables for culture, role, and geography were all significant; these were not qualified by 2- or 3-way interactions (Table 3). As predicted, all five beliefs were significantly stronger for Mapuche than non-Mapuche participants. Only one belief was significantly different for role: parents valued a calm child significantly more than teachers. Two beliefs were significantly different for location. Surprisingly, those in urban settings believed in the importance of Kumeche and nature as a regulator significantly more than those who dwell in rural areas.

**TABLE 3:** Univariate comparisons, significant effects, and combined effects for the Mapuche-generated scales.

	***M* (*SD*)**		***df***	***F***	**Partial η^2^**
**Culture (combined effect)**	**Mapuche**	**Non-Mapuche**	**5, 266**	2.41*	**0.049**
Control of fear	4.13 (1.14)	3.79 (1.13)	1, 270	7.08**	0.027
Being a calm child	4.07 (1.17)	3.82 (0.98)	1, 270	7.47**	0.029
Kumeche	4.22 (1.30)	4.18 (1.01)	1, 270	6.60*	0.025
Learning through observation	4.57 (1.24)	4.33 (0.95)	1, 270	7.89**	0.030
Regulation through nature	4.57 (1.30)	4.31 (1.12)	1, 270	9.40**	0.036

**Role**	**Parent**	**Teacher**	**5, 266**	2.79*	**0.053**

Being a calm child	4.03 (1.09)	3.72 (0.98)	1, 270	5.73**	0.022

**Location**	**Urban**	**Rural**	**5, 266**	2.56*	**0.049**

Kumeche	4.32 (1.01)	3.97 (1.35)	1, 270	7.57**	0.026
Regulation through nature	4.47 (1.10)	4.29 (1.44)	1, 270	5.00*	0.019

Participants responded to items using a Likert scale ranging from one (totally disagree) to six (totally agree). Pillai’s Trace reported. *p < 0.05, **p < 0.01.

## Discussion

Overall, we found similarities across cultures as well as differences in emotional beliefs about children embedded within cultural norms and values. Similarities across Mapuche and non-Mapuche parents and teachers were found for beliefs about children’s emotional autonomy and efficacy in controlling and regulating emotion, and the belief that when emotions are not in check, even positive emotions can have costly consequences.

Differences in emotion beliefs were shown by the stronger belief of the Mapuche compared to non-Mapuche that children can and should outgrow feeling fear and should also be calm, quiet children, themes which are consistent with previous findings using very different methodologies (Oertwig et al., 2019). These consistent findings from both qualitative interviews and explicit measurement via questionnaires, as in the present study, fit well with the unique history of the Mapuche. We also note that, although the differences are significant, they are not starkly different, suggesting cross-fertilization of beliefs across the culturally different communities that have integrated somewhat over time.

With regard to the emotion socialization pathways supported by these beliefs, two findings can be highlighted: Mapuche traditions of listening to elders’ storytelling and watching elders may be important to distinguish from the greater emphasis on verbal discussion that seems to characterize United States cultural contexts or by “observing and pitching in” in Central American cultural contexts (Rogoff, 2014). Another important contribution from the Mapuche tradition is the belief that nature provides an important way of regulating children’s emotions; environmental psychology research demonstrates that this belief is well-warranted (Bowler et al., 2010; Kondo et al., 2018; Pasanen et al., 2018). Learning about the beliefs that specific cultures convey regarding emotion regulation can enlarge the emotion socialization toolbox for all, and suggest specific and important additions to the four basic strategies suggested 20 years ago (Eisenberg et al., 1998a).

Parental and teacher beliefs were very similar, showing only two differences: Mapuche parents reported believing that children can control emotions more than Mapuche teachers, and parents in general more strongly valued a calm child compared to teachers. These findings do support the notion that Mapuche teachers have more of an occidental, Eurocentric perspective similar to non-Mapuche teachers and compared to Mapuche parents. They also will have had greater familiarity with non-Mapuche children who are not socialized with the same values for being calm (as shown in our data), and are also in classrooms with many children who thus have increased opportunities for emotional contagion.

Geographical location appeared important in three ways. The interaction with culture and location suggested that the Mapuche, whether rural or urban, do value knowing what their children are feeling, but rural non-Mapuche had less belief that it was important to know children’s feelings. Perhaps the dangers associated with living in a city may lead urban participants to feel the need to know more about children’s lives, regardless of their cultural background. Additionally, those in urban settings believed in the importance of Kumeche (attentiveness, compassion, kindness) and nature as a regulator significantly more than those in rural areas. These main effects were surprising to us. It may be that life without much support for solidarity and community structure and lack of nature constantly surrounding the family may be missed by urban families. At the same time, rural families may not hold such strong beliefs for what is present in their lives and may take for granted the importance of nature as an emotion regulator.

We also found that the differences between the Mapuche and non-Mapuche for emotion beliefs generated by the Mapuche, although significant, were not as great as we expected. We note the challenge of questionnaire research in populations which are not that familiar with questionnaire assessment and who come from more of a conversational, story-telling tradition. In this way, our results may be more conservative than true to the differences across the cultures. Alternatively, that many Mapuche and non-Mapuche live in the same communities, and have for some time, may suggest cross-fertilization of thinking as well as continued variation in the depth of acceptance of these beliefs.

Because most children in Chile are taught by non-Mapuche teachers, results highlight some of the challenges facing children being taught by non-culturally connected teachers. For example, non-Mapuche teachers might think that quiet, calm children are not showing sufficient enthusiasm or are not paying attention to lessons. Further, when Mapuche children need to calm themselves, their teachers may not perceive the importance of letting children spend time outside in nature. These results also suggest new, distinctive beliefs about emotion, which, now that they have been identified, can be assessed in other countries as well. This is important in that ideas about emotion “residue” found in Indian culture were also surprisingly prevalent in the United States once researchers thought to study them in the United States (Savani et al., 2011). In the same way, beliefs about the value of nature may be more prevalent in the United States than previously imagined. Studying socializers’ attention to different emotional beliefs, such as the value and ability to be unafraid; to be attentive to others, and to watch and listen to elders (thereby showing respect for others); and to be in nature, with its perceived centrality for emotion regulation, may all be useful in enlarging socializers’ perspectives regarding the beliefs that they want to inculcate in the young of their own cultures.

Finally, we note some of the limitations of the study. First, the Mapuche and non-Mapuche participants were recruited via a snowball method, and so may be more highly educated and/or economically privileged than their counterparts who did not have a college student in their friendship or family networks. We do not know if this would increase or decrease the strength of our findings, but we do note the problem. We also measured beliefs via questionnaires, which is one of many methods, and requires participants to fit their beliefs into explicitly stated questions which might not have sufficient precision to their own unique beliefs, or might make them wonder what the “right” answer might be. However, a strength of the study is that the beliefs we asked were initially generated from long conversations in which implicit as well as explicit beliefs could be revealed, and these were utilized in questionnaire construction. Further, we note that for both the United States- and the Mapuche-generated beliefs, the mean scores hover slightly above the middle for most scales, indicating mild agreement with the beliefs. Exceptions were the relatively high agreement with the importance of knowing what children are feeling (except for rural, non-Mapuche), and the relatively low agreement regarding children being autonomous. There was also good variability for all beliefs. These results, along with the internal reliability indices, suggest that the beliefs we included are recognized and have meaning for the people responding to them. We also do not know the degree to which participants resonated with these beliefs in principle, but not in actuality. That is, there is a potentially large difference between what people say they believe and then demonstrate in their actions.

Of course, there are many emotion-related beliefs, and this set of 11 beliefs is just a subset of what is possible to explore. Nevertheless, we hope that by identifying both similarities and differences between the cultures, we have highlighted particular beliefs worth exploring in these cultural contexts as well as others.

## Data Availability Statement

The datasets generated for this study are available on request to the corresponding author.

## Ethics Statement

The study was reviewed and approved by the Comité de Ética de la Investigación de la, Universidad Católica de Temuco. The participants provided their written informed consent to participate in this study.

## Author Contributions

Based on qualitative interviews: AH and ER wrote items for the Mapuche-generated scales. AH advised ER re: PBACE scales and items, supervised the analyses, and wrote and edited much of the manuscript. DO analyzed the data, wrote the “Results” section, created the tables, and contributed to the “Introduction,” “Materials and Methods,” and “Discussion.” ER developed the relationships with Mapuche that enabled us to collect the data, conceptualized the study, wrote items for the Mapuche-generated scales (with AH), supervised the translation process (both within Spanish and across to English), supervised the data collection, wrote the portions of the “Materials and Methods” and “Introduction’ sections, and helped to revise the “Discussion” section.

## Conflict of Interest

The authors declare that the research was conducted in the absence of any commercial or financial relationships that could be construed as a potential conflict of interest.
