# Supplementary material for: Beliefs About Children’s Emotions in Chile
Source: Front Psychol. 2020 Jan 30;11:34. doi: 10.3389/fpsyg.2020.00034 (PMC7002361; doi:10.3389/fpsyg.2020.00034)
Supplement: Supplementary file 1 [file Table_1.pdf]

Cree-emoción Cuestionario

Estas declaraciones expresan diferentes creencias sobre el desarrollo emocional de los niños y el rol que cumplen los padres en ayudarles a manejar sus emociones. Por favor lea cada una de las afirmaciones y marque el número que mejor representa cuánto usted está de acuerdo o en desacuerdo con ellas. Indique su respuesta en la columna titulada "Respuesta."

1<br>Totalmente en Desacuerdo	2<br>Algo en Desacuerdo	3<br>Un poco en Desacuerdo	4<br>Un poco en Acuerdo	5<br>Algo en Acuerdo	6<br>Totalmente en Acuerdo

Como las capacidades de los niños se desarrollan a lo largo del tiempo, por favor marque la edad que tiene el niño con el que usted está familiarizado (entre 4 a 10 años), y responda de acuerdo a esa edad (indicar esa edad aproximada acá_____).

N°	Ítem	Respuesta
1	Los niños usan sus emociones para manipular a otros.	
2	Usualmente es mejor dejar al niño que maneje su tristeza por sí solo.	
3	Es útil para los niños sentir enojo a veces.	
4	Los padres no tienen que saber acerca de los sentimientos de los niños.	
5	Los niños pueden controlar lo que muestran en sus rostros.	
6	Usualmente es mejor dejar al niño que maneje sus sentimientos negativos por sí solo	
7	Los niños pueden controlar cómo expresan sus sentimientos.	
8	Los niños tienden a actuar tristes o enojados solo para salirse con la suya.	
9	Es saludable que los niños expresen su rabia.	
10	Los niños a menudo lloran solo para obtener atención.	
11	Los padres deberían alentar a sus niños a decirles todo lo que están sintiendo.	
12	Aun cuando los niños están muy felices ellos pueden controlar su expresión emocional.	
13	Los niños pueden controlar sus emociones.	
14	Es importante para los niños decirle a sus padres todo lo que están sintiendo.	
15	Cuando los niños están muy felices, pueden salirse de control.	
16	Mucha alegría puede hacerle difícil a un niño que entienda a otros.	
17	Cuando los niños están enojados, necesitan encontrar sus propias formas de resolver la situación.	
18	Los niños necesitan aprender no reaccionar con miedo.	
19	Los niños que sienten emociones fuertemente son más propensos a problemas en la vida.	
20	Los niños a veces actúan tristes solo para obtener atención.	
21	Controlar el miedo es una característica distintiva de un niño estable.	
22	Los espíritus de la tierra pueden ayudar a tranquilizar emocionalmente al niño.	
23	Cuando un niño siente tristeza, el buscar un lugar tranquilo fuera de la casa puede ser una buena estrategia para sentirse mejor.	

24	Los niños se vuelven regulados emocionalmente cuando escuchan el consejo de sus padres.	
25	Los niños deben controlar sus emociones con el fin de centrar su atención.	
26	Un niño tranquilo tiene la regulación emocional mejor que un niño hablador.	
27	El contacto con la naturaleza ayuda a los niños a regular su emoción cuando sienten miedo.	
28	Estar tranquilo es clave para el control de las emociones.	
29	Es importante que los niños respeten, pero no teman la naturaleza.	
30	Cuando los niños aprenden a escuchar los consejos de los adultos también aprenden cómo controlar sus emociones.	
31	Evitar sentir miedo hace al niño más seguro de sí mismo.	
32	Un niño que habla mucho de sus emociones es más estable emocionalmente.	
33	Un niño sabio puede regular sus emociones.	
34	Los niños aprenden el respeto observando cómo sus padres muestran respeto a sus abuelos.	
35	Los padres pueden ayudar a un niño a obtener el control de sus emociones conectándolos con la naturaleza.	
36	Los niños aprenden a regularse emocionalmente escuchando a los adultos.	
37	Un niño puede controlar mejor sus emociones cuando está tranquilo que cuando está hablando.	
38	Los niños deben estar atentos a las necesidades de los otros.	
39	En silencio escuchando conversaciones entre adultos, y las historias de los abuelos, es una buena manera de aprender cómo comportarse.	
40	La tierra [arboles, ríos, etc.] ayuda a los niños a regular su tristeza.	
41	Parte del crecimiento es aprender a no sentir miedo.	
42	La naturaleza puede ayudar a los niños regular sus expresiones emocionales.	
43	Enfrentar al miedo ayuda al niño ser más resistente.	
44	Cuando los niños están muy enojados pueden controlar lo que demuestran a otros.	
45	Los niños necesitan aprender a no tener miedo.	
46	Estar tranquilo puede ayudar al niño a ser más consciente de las emociones de los demás.	
47	Expresar la rabia es una buena forma para que los deseos y opiniones de un niño sean conocidos por los demás.	
48	Ir a escuchar un rio puede ser bueno para tranquilizar un niño.	
49	Los niños pueden no enfocarse en sus compromisos si se sientan demasiado felices.	
50	Desde pequeño los niños debieran ser capaces de ser buenos anfitriones.	
51	Desde pequeño los niños debieran mostrar su cariño a sus abuelos.	

TAMBIEN: necesitamos algunos antecedentes que permitirán realizar un mejor análisis.

Edad: Género= Hombre / mujer Nivel en el que enseña:
Rol: [1] Padre/madre [2] Profesor(a)

Es importante para nosotros saber si Ud. pertenece a algún pueblo originario: si / no
Si contestó afirmativamente indique a cuál: ______________________________
¡GRACIAS!

Dimensiones e ítems del instrumento

Escala	Nº ítems	Ítems
**Dimensión Original**		
1. Autonomía	3	2;6;17
2. Conocimiento parental	3	4(Inverso);11;14
3. Control	5	5;7;12;13;44
4. Costo Positividad	4	15;16;19;49
5. Manipulación	4	1;8;10;20
6. Valor de la rabia	3	3;9;47
**Nuevas dimensiones**		
7. Control del Miedo	6	18;21;31;41;43;45
8. Kumeche	4	33;38;50;51
9. Padres	4	24;34;36;39
10. Tierra	8	22;23;27;29;35;40;42;48
11. Tranquilidad	7	25;26;28;30;32;37;46

Riquelme, E. H., Miranda, E., Halberstadt, A. G. (in press). Creencias de los adultos sobre la emoción de los niños: una propuesta de exploración en contextos de diversidad cultural (Adults' beliefs about the emotion of children: A proposal for exploration in contexts of cultural diversity). *Estudios Pedagógicos.*
